# An Immunochemical Approach to Detect the Quorum Sensing-Regulated Virulence Factor 2-Heptyl-4-Quinoline N-Oxide (HQNO) Produced by Pseudomonas aeruginosa Clinical Isolates

**DOI:** 10.1128/spectrum.01073-21

**Published:** 2022-07-25

**Authors:** Enrique J. Montagut, Juan Raya, M.-Teresa Martin-Gomez, Lluïsa Vilaplana, Barbara Rodriguez-Urretavizcaya, M.-Pilar Marco

**Affiliations:** a Nanobiotechnology for Diagnostics (Nb4D), Department of Surfactants and Nanotechnology, Institute for Advanced Chemistry of Catalonia (IQAC) of the Spanish Council for Scientific Research (CSIC), Barcelona, Spain; b CIBER de Bioingeniería, Biomateriales, y Nanomedicina (CIBER-BBN), Barcelona, Spain; c Microbiology Department, Vall d’Hebron University Hospital (VHUH), Barcelona, Spain; d Genetics and Microbiology Department, Universitat Autònoma de Barcelona (UAB), Barcelona, Spain; Technical University of Denmark

**Keywords:** *Pseudomonas aeruginosa*, quorum sensing, virulence, diagnostic, antibodies, immunoassay, HQNO-2-heptyl-4-quinoline N-oxide, MvfR, PqsR, virulence factors

## Abstract

Understanding quorum sensing (QS) and its role in the development of pathogenesis may provide new avenues for diagnosing, surveillance, and treatment of infectious diseases. For this purpose, the availability of reliable and efficient analytical diagnostic tools suitable to specifically detect and quantify these essential QS small molecules and QS regulated virulence factors is crucial. Here, we reported the development and evaluation of antibodies and an enzyme-linked immunosorbent assay (ELISA) for HQNO (2-heptyl-4-quinoline N-oxide), a QS product of the PqsR system, which has been found to act as a major virulence factor that interferes with the growth of other microorganisms. Despite the nonimmunogenic character of HQNO, the antibodies produced showed high avidity and the microplate-based ELISA developed could detect HQNO in the low nM range. Hence, a limit of detection (LOD) of 0.60 ± 0.13 nM had been reached in Müeller Hinton (MH) broth, which was below previously reported levels using sophisticated equipment based on liquid chromatography coupled to mass spectrometry. The HQNO profile of release of different Pseudomonas aeruginosa clinical isolates analyzed using this ELISA showed significant differences depending on whether the clinical isolates belonged to patients with acute or chronic infections. These data point to the possibility of using HQNO as a specific biomarker to diagnose P. aeruginosa infections and for patient surveillance. Considering the role of HQNO in inhibiting the growth of coinfecting bacteria, the present ELISA will allow the investigation of these complex bacterial interactions underlying infections.

**IMPORTANCE** Bacteria use quorum sensing (QS) as a communication mechanism that releases small signaling molecules which allow synchronizing a series of activities involved in the pathogenesis, such as the biosynthesis of virulence factors or the regulation of growth of other bacterial species. HQNO is a metabolite of the Pseudomonas aeruginosa-specific QS signaling molecule PQS (Pseudomonas quinolone signal). In this work, the development of highly specific antibodies and an immunochemical diagnostic technology (ELISA) for the detection and quantification of HQNO was reported. The ELISA allowed profiling of the release of HQNO by clinical bacterial isolates, showing its potential value for diagnosing and surveillance of P. aeruginosa infections. Moreover, the antibodies and the ELISA reported here may contribute to the knowledge of other underlying conditions related to the pathology, such as the role of the interactions with other bacteria of a particular microbiota environment.

## INTRODUCTION

Pseudomonas aeruginosa is a ubiquitous Gram-negative bacteria able to colonize all types of living entities due to its metabolic versatility and adaptability. In humans, it behaves as an opportunistic pathogen, causing a broad spectrum of life-threatening acute and chronic infections in the urinary tract, burn and wound injuries, ventilator-associated pneumonia, and bacteremia (1). The broad set of pathogenic and survival mechanisms shown by this bacterium makes it one of the most commonly isolated microorganisms in clinical settings. Additionally, P. aeruginosa is listed in the 2019 Antimicrobial Resistance Threats Report (2) of the CDC (Centers for Disease Control and Prevention) as a serious threat because it showed antimicrobial resistance (AMR) to multiple drugs causing critical health care and economic burden worldwide (3–5). More than 2.8 million antibiotic-resistant infections occur in the US each year and more than 35,000 people die as result. A recent European Center for Disease Control and Prevention (ECDC) study (6) on the health burden of antibiotic resistance estimated that about 33,000 people die each year in the EU/European Union/European Economic Area (EEA) as a direct consequence of an infection due to bacteria resistant to antibiotics. The actual cost of antimicrobial resistance (AMR) to human health is highly variable and difficult to calculate. However, it has been estimated a global economic price of €90 billion by 2050 (7).

Multidrug-resistant P. aeruginosa predominantly affects patients with a depressed immune system and can be particularly dangerous for patients with chronic lung diseases. Hence, in 2017, multidrug-resistant P. aeruginosa caused an estimated 32,600 infections among hospitalized patients and 2,700 estimated deaths in the United States. P. aeruginosa is the origin of recurrent infections in patients with cystic fibrosis (CF) (8), the most common lethal genetic disorder in Caucasians. For instance, P. aeruginosa prevalence in patients with CF ranges from approximately 25% for children under 5 years old to 80% for adults from 25 to 34 years of age (9). Additionally, it has been estimated that a median survival at the birth of 46 years in males and 41 years in females is critically determined by the infections that became chronic during their lifetime (10). Fast identification of the pathogen is crucial for the proper management of these patients to provide them with adequate and specific treatments on time and avoid the transition from acute to persistent infection (11).

Current diagnostic methods are based on the analysis of metabolic and morphologic characteristics of pure isolated bacteria. This type of analysis can take up to 72 h and several problems can arise from the procedure, where many bacteria can be missed and/or misidentified (12). Developing new diagnostic strategies that fill the lack of sensitivity and specificity while delivering rapid results in identifying the causative agent is of utmost importance. Many techniques have emerged aiming to substitute or complement the standard methods. For instance, surface-enhanced Raman spectroscopy has been successfully applied for the detection of infectious pathogens and polymerase chain reaction (PCR) is a habitual technique used in microbiology hospital departments (13–16). MALDI-TOF-MS (matrix-assisted laser desorption ionization-time of flight mass spectrometry) has also become an important tool for clinical microbiologists, providing characteristic bacteria protein profiles (17). Nonetheless, these techniques require expensive equipment, tedious extractions, and purification procedures and still need culture pre-enrichment steps to accomplish the necessary sensitivity (18, 19).

The bacterial communication system known as quorum sensing (QS) has attracted the attention of many researchers as a target for therapy and diagnostics of infectious diseases (20, 21). This cell-density-based communication system relies on the release of low molecular weight signals that control the genetic expression of relevant bacterial common goods (22). These quorum sensing molecules induce their biosynthesis and when the local concentration reaches a specific threshold, it triggers the transcription of genes related to several pathogenic mechanisms, such as the secretion of virulence factors and biofilm synthesis (23). P. aeruginosa QS is composed of four interconnected systems. Each system possesses a characteristic autoinducer (AI), such as homoserine lactones (*las* and *rhl* systems) or alkyl quinolones (*PqsR* system) (24). The 2-heptyl-3-hydroxy-4 (1H)-quinolone (PQS) is the one that has been reported to bind with higher affinity to the PqsR communication system, which performs many bacterial functions besides its signaling activity (25). Although PQS has focused the vast majority of studies, P. aeruginosa produces other quinolones that also carry out crucial processes for bacterial survival. Between them, 2-heptyl-4-quinoline N-oxide (HQNO) is one of the most abundant alkyl quinolones/quinolines produced by this pathogen. This metabolite acts as an inhibitor of the respiratory electron chain, and it is considered a major virulence factor, showing the capability of damaging host cells and several microorganisms (26). It allows P. aeruginosa to subjugate Staphylococcus spp., forcing them to persist as small colony variants (SCVs) (27). HQNO reduces S. aureus metabolism through inhibition of the cytochrome systems and interestingly induces multidrug bactericidal tolerance (28). The bacterial interspecies interaction is frequent in diseases, such as CF, where S. aureus is predominantly present from birth and is progressively displaced and outcompeted by P. aeruginosa during the CF patient's lifetime (29, 30). An isolated bacterium is rarely detected when a microbiological analysis of a sample from a CF patient is performed. Hence, studying coinfections and the interactions happening in the host is fundamental for a complete understanding of the disease's underlying conditions. The development of new techniques for the quantification of the metabolites that determine this kind of interaction would shed light on the complex pathogenic mechanisms and etiology of bacterial infections.

There are few reported studies in the literature with respect to HQNO. However, it has already been detected in culture samples using different techniques (31–34). This metabolite and other quinolones have also been found in clinical samples of CF patients by liquid chromatography-mass spectrometry (LC-MS), pointing to the potential of the quorum sensing molecules (QSMs) as biomarkers of infection (35, 36). The evaluation of QSMs might be useful for the prediction of exacerbation periods, to monitor the state of the disease, or study the relevance of interspecies interaction in the host during an infectious process. Nevertheless, further well-designed clinical studies have to be conducted to validate this hypothesis and to exploit their full potential diagnostic and prognostic value. For this purpose, the availability of reliable high-throughput diagnostics technologies able to quantify these QSMs is crucial.

For several years, we focused our attention on QS, seeking to get knowledge on QS's role in developing pathogenesis. With this aim, we reported the development of antibodies and immunochemical tools for other QSMs and virulence factors (37–40), which have shown great potential for therapeutic and diagnostic applications, respectively. HQNO is involved in crucial aspects related to P. aeruginosa virulence driving interspecies interaction and being partially responsible for the incidence and prevalence of this pathogen in respiratory infections. For this reason, we report the development of specific antibodies for HQNO and their use to establish an immunochemical assay for the quantification of this molecule in complex biological and clinical samples, such as those from patients undergoing pulmonary infections.

## RESULTS AND DISCUSSION

As stated in the introduction HQNO is a valuable P. aeruginosa quinolone-type QSM, acting also as a virulence factor and regulating the growth of other coexistent bacterial species, such as S. aureus. Because of its relevance, continuous monitoring of its profile of release could provide interesting information related to the pathogenesis and disease progression and in addition to using it for diagnostic purposes. Immunochemical analytical methods are exceptional tools to detect and quantify a wide variety of biomarkers in complex biological samples with high efficiency and reliability. The key aspect is the availability of high-quality antibodies toward the biomarker targets of interest. Thus, in this case, we focused on the development of antibodies against HQNO. Because of its small size (<500 Da), HQNO was not able to elicit an immune response, therefore precluding direct production of antibodies. Antibody production requires linking these molecules to a higher size biomacromolecule able to strongly activate the immune system in selected experimental animals. With this purpose, a hapten mimicking the original chemical structure of the target was rationally designed incorporating a linker, or spacer arm, with a chemical functional group suitable for covalent attachment to a biomacromolecule, to rend the immunogen. It is of extraordinary importance that this approach did not block important antigenic epitopes of the molecule. With this criterion in mind, HQNO hapten was designed to preserve the functional groups at positions.

**FIG 1:**
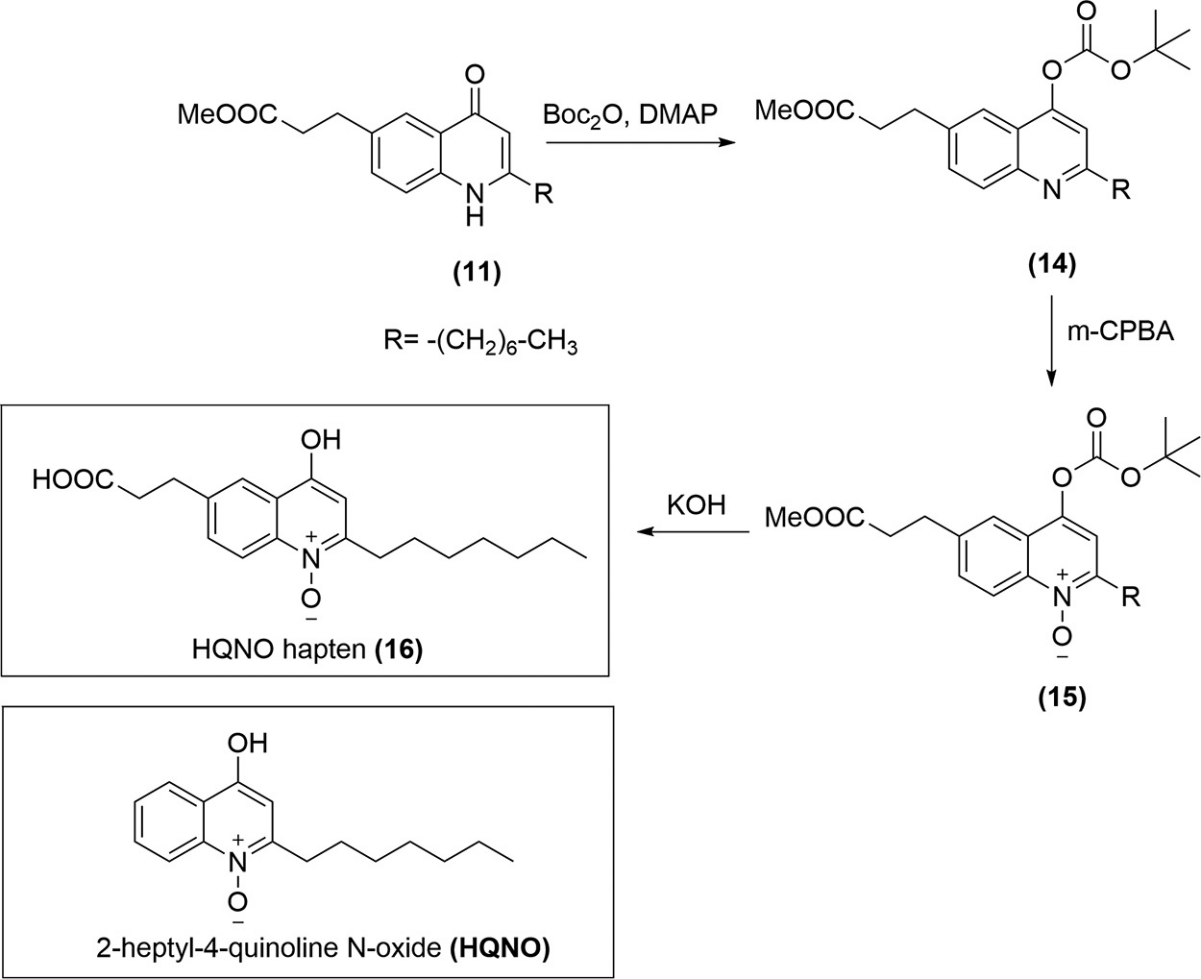
Synthetic scheme for the synthesis of HQNO hapten (16), analogous to 2-heptyl-4-quinoline N-oxide of the *PqsR* system from P. aeruginosa. The hapten was synthesized through a three steps synthetic pathway from methyl 3-(2-heptyl-4-oxo-1,4-dihydroquinolin-6-yl) propanoate.

N-1 and C-2 to C-4 are unaltered (see Fig. 1) because these epitopes were considered to be the most characteristic ones of the molecule, with a capability to establish strong noncovalent interactions with the antibodies that would be produced (41, 42). For this purpose, the spacer arm of the hapten was placed at C-6, maximizing in that way the recognition of the most reactive epitopes while minimally altering the electronic structure of the original molecule. The spacer arm ended with a carboxylic acid group that allowed its reaction to the amino groups of the lysine residues of the protein through orthogonal chemistry.

The synthesis of the hapten followed the same strategy as that described by Woschek et al. (43) but using methyl 3-(2-heptyl-4-oxo-1,4-dihydroquinolin-6-yl) propanoate (11, 39) (Fig. 1) as starting material. To prevent undesired reactions due to the high nucleophilicity of the position C-3 and the electrophilic character of C-4 due to the carbonyl, blocking off the corresponding tautomer was first addressed by protecting this carbonyl group with di-tert-butyl dicarbonate and a catalytic amount of N,N-dimethylpyridin-4-amine (DMAP), obtaining the intermediate (14) with an 88% yield. Afterward, the oxidation of the nitrogen to obtain the characteristic N-oxide functional group was achieved by reaction with meta-chloroperoxybenzoic acid (m-CPBA), obtaining the intermediate (15) in 92% yield. Finally, the desired HQNO hapten (16) was obtained by simultaneous deprotection of the carbonyl and the carboxylic acid using a degassed solution of potassium hydroxide (KOH). The overall yield of the three synthetic steps was 62%.

Bioconjugation of the hapten (16) was carried out generating a mixed anhydride with isobutyl chloroformate and a hindered base, which reacted rapidly with the amino groups of the lysine residues of the proteins. The HQNO-KLH bioconjugate was used to raise polyclonal antibodies in female New Zealand white rabbits obtaining the antisera named As388, As389, and As390. The avidity of these antisera for the BSA conjugates (HQNO-BSA and HHQ-BSA [40]) was assessed through two-dimensional non-competitive indirect ELISAs, which allowed the determination of the suitable concentrations of these immunoreagents for developing competitive assays.

Competitive immunoassays were only obtained under heterologous conditions using HHQ-BSA as a bioconjugate competitor. The best antisera/bioconjugate combination was selected based on the maximum absorbance achieved, the background noise, the IC_50_ parameter, and the slope of the calibration curves (Table S2). Thus, As389/HHQ-BSA was finally elected for further studies about the performance under different physicochemical conditions such as pH, ionic strength, the concentration of a nonionic surfactant, presence of an organic solvent, and the effect of the incubation time. As can be observed in Fig. S1, the pH and the conductivity of the assay buffer and the concentration of the surfactant Tween 20 were the factors that most affected the assay. Regarding pH, the best detectability was accomplished at pH values between 5.5 and 6.5, performing better under acidic than under basic conditions. Thus, at pH 4.5 the assay still could be used with IC_50_ values slightly higher (38 nM versus 23 nM at pH 6.5) while at pH 7.5 the IC_50_ value was 52 nM and pH 8.5 raised to 93 nM. The maximum absorbance of the assay reached the higher value at pH 6.5, while below this pH decreased significantly. With respect to the concentration of Tween 20 in the buffer, the best features were obtained in the absence of surfactant (IC_50_, 19 nM), observing a decrease in the detectability already at 0.01% (IC_50_, 43 nM) with raised dramatically when increasing the concentrations of the nonionic surfactant. On the other hand, higher conductivities favored the detectability, although the maximum absorbance and the slope proportionally worsened compromising the performance of the assay. A conductivity of 15 mS cm^−1^ was selected as a compromise between the maximum signal of the assay and detectability for which the slope of the calibration curve was also acceptable (−0.75 ± 0.04) for proper quantification of the target. The competition time was found to be optimal at 30 min, and there was not a significant improvement in the assay features when adding a preincubation step.

**FIG 2:**
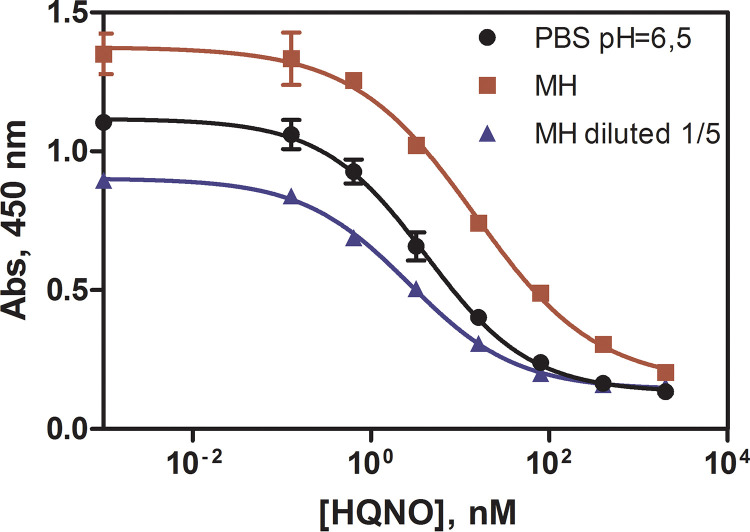
Calibration curves of the As389/HHQ-BSA ELISA for the detection of HQNO in buffer (PBS-6.5) and 1/5 diluted MH broth, under the conditions established (Table 1). Each calibration point was measured in triplicates on the same ELISA plate and the results showed the average and standard deviation of analysis made on three different days.

After these studies, the conditions established for the As389/HHQ-BSA ELISA improved significantly the detectability of the assay and involved the use of PBS-6.5 as the assay buffer. The calibration curve recorded under these conditions is shown in Fig. 2. HQNO could be detected with a LOD of 0.27 ± 0.09 nM, an IC_50_ of 4.20 ± 0.86 nM, and a dynamic range compressed between 0.72 ± 0.18 to 26.71 ± 0.96 nM (Table 1). The detectability achieved was far below the values reported in bacterial cultures (μM range) (28, 33, 34, 44) and the same range as those found in clinical samples (nM range) (35, 36). Usually, HQNO quantification is made by LC-MS that despite their robustness requires tedious extractions and intermediate preconcentration steps to achieve the required detectability (35, 36, 45), while the ELISA here reported may directly achieve comparable performance.

**TABLE 1:** Features of the As389/HHQ-BSA ELISA for the detection of HQNO

Metric	PBST^*a*^	MH	MH diluted 1/5
A_min_	0.09 ± 0.04	0.12 ± 0.01	0.09 ± 0.02
A_max_	1.08 ± 0.06	1.32 ± 0.08	0.85 ± 0.01
Slope	−0.75 ± 0.04	−0.64 ± 0.03	−0.72 ± 0.06
IC_50_	4.20 ± 0.86	14.65 ± 2.21	2.71 ± 0.04 13.55 ± 0.20)
Dynamic range	0.72 ± 0.18 to 26.71 ± 0.96	1.84 ± 0.38 to 105.37 ± 5.98	0.41 ± 0.10 to 17.04 ± 1.08
LOD	0.27 ± 0.09	0.60 ± 0.13	0.15 ± 0.05 (0.75 ± 0.25)
R^2^	0.987 ± 0.013	0.998 ± 0.001	0.990 ± 0.004

a The concentration of BSA conjugate and dilution used in the assay run in buffer (PBS-6.5) was 0.25 μg mL^−1^ and 1/8000, respectively. In the case of the assay run in MH or MH diluted 1/5 the concentration of BSA conjugate and As dilution were 0.25 μg mL^−1^ and 1/8000, respectively. The parameters and features of the MH 1/5 curve correspond and refer to the values in the diluted sample and brackets are calculated for the corresponding IC_50_ and LOD in MH culture media. The concentrations are expressed in nM and the data shown correspond to the average of 3 different days using at least 2 well/replicates per concentration.

**FIG 3:**
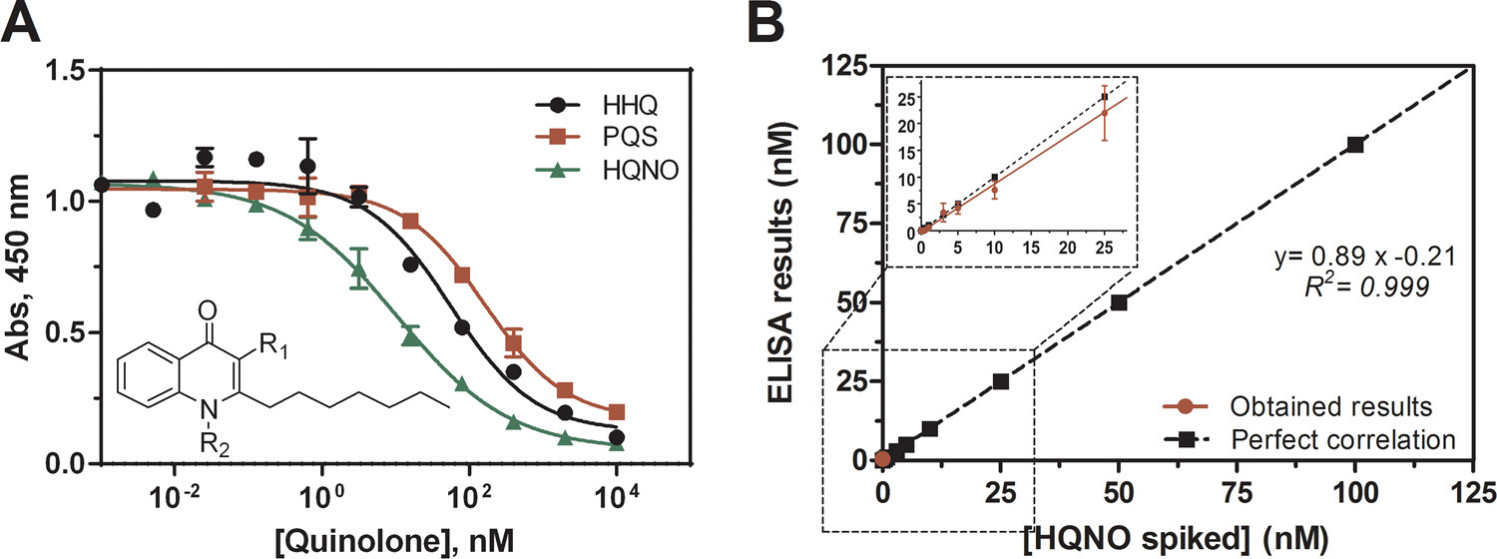
(A) Cross-reactivity study using the *PqsR* quorum sensing metabolites HHQ, PQS, and HQNO in buffer under the aforementioned conditions for As389/HHQ-BSA ELISA. Calculated cross-reactivity was 19% for HHQ (IC_50_ = 56.0 nM) and 7% for PQS (IC_50_ = 162.5 nM). HHQ: R1 = −H, R2 = −H; PQS: R1 = −OH, R2 = −H; HQNO: R1 = −H, R2 = OH. (B) Results from the accuracy study. The graph showed the linear regression analysis of the HQNO concentration spiked in MH broth and the concentration measured with the As389/HHQ-BSA ELISA developed. Assays were run in diluted MH culture media 1/5 using PBS-6.5. Each calibration point was measured in triplicates on the same ELISA plate and the results showed the average and standard deviation of analysis made on three different days.

P. aeruginosa produced a wide variety of quorum sensing molecules, among which quinolones are the main signals and metabolites from the *PqsR* communication system. Despite the similarities in their chemical structures, PQS, HHQ, and HQNO have different roles and functions. Because there was a high chance of finding these quinolones secreted simultaneously during an infection or in culture, it was necessary to assess the assay specificity toward these other QSMs. The results of these studies showed that the As389/HQNO-BSA ELISA recognized HQNO to a greater extent with low interferences from the PQS (7% cross-reactivity [CR]) and HHQ (19% CR) (Fig. 3A). Presumably, HHQ was better recognized because of the lack of functionalization at the C-3 position of the quinolone core, as in HQNO. As stated above the immunizing hapten was designed to maximize recognition of this molecule moiety. These percentages of CR may play a role during the quantification of HQNO and, therefore, should be taken into account, and this is the reason the concentration of HQNO measured in biological samples was expressed as immunoreactivity (IR) equivalents in this work. The percentage of CR of other structurally related substances quinolone-type antibiotics (ciprofloxacin and norfloxacin) and structurally and nonstructurally related QS molecules (DHQ, 2,4-dihydroxyquinoline; 2-AA, 2-aminoacetophenone; IQS, 2-[2-hydroxyphenyl]-thiazole-4-carbaldehyde) as well as virulence factors (pyocyanin [PYO]), which could also be eventually present on a clinical sample, was found to be negligible (<0.01%) as shown in Table 2.

**TABLE 2:** Half maximal inhibitory concentration (IC_50_) of the As389/HHQ-BSA ELISA using as analytes HHQ, PQS, HQNO, PYO, IQS, and the quinolone-type antibiotics ciprofloxacin and norfloxacin

Quinolone	IC_50_^*a*^	C.R.^*b*^ (%)
HHQ	56.0	19
PQS	162.5	7
HQNO	10.7	100
PYO	-	<0.01%
IQS	-	<0.01%
DHQ	-	<0.01%
2-AA	-	<0.01%
Ciprofloxacin	-	<0.01%
Norfloxacin	-	<0.01%

a Dashes indicates that there is no cross-reactivity when testing that combination.

b The percentages of cross-reactivity (C.R.) were calculated following the equation: CR (%) = IC_50_(cross reactant)/IC_50_(analyte) × 100.

With the knowledge regarding the performance of the As389/HHQ-BSA ELISA, we addressed the investigation of the HQNO release kinetics of P. aeruginosa on a culture medium, such as Müeller Hinton (MH). Previously, the potential nonspecific interferences in this complex biological matrix were assessed by building calibration curves in MH diluted several times with PBS-6.5 (Fig. S2). As shown in Fig. 2 and Table 1, the assay performed well in undiluted MH broth, although compared to the assay run in buffer, a slight increase of the maximum signal with a concomitant decrease of the assay detectability (IC_50_ 4.2 in buffer versus 14.6 in MH) could be observed. This effect diminished when diluting the MH media with the assay buffer reaching better features at 1/5 dilution factor (IC_50_ 2.7 nM, 13.5 nM considering the dilution of the culture media with the assay buffer). Although detectability was possible to directly measure HQNO in the culture medium without any dilution, we used a 1/5 dilution in further studies to prevent or reduce potential nonspecific interferences caused by the release of other bacterial exoproducts. The slight decrease in detectability with respect to the assay run in buffer did not compromise the usefulness of the assay because, as previously mentioned, HQNO levels have been found in the μM range (31, 33, 34) in culture medium.

The accuracy of the As389/HQNO-BSA ELISA in 1/5 diluted MH was evaluated by preparing blind samples spiked with HQNO concentrations inside and outside the dynamic range. These samples were then measured in triplicates on the same ELISA microplate and the same experiments were repeated on three different days. As shown in Fig. 3B, the correlation between the spiked and the measured concentration was quite good with a slope of 0.89 ± 0.01 nM and a regression coefficient of 0.999.

The precision of the assay was also assessed by calculating the percentage of coefficient of variation within the same plate (intraplate variation), between diverse plates (interplate variation), and on different days (Table 3). These experiments were done at three different levels of concentrations (low IC_80_, medium IC_50_, and high IC_20_). In general, the percentage of CV remained below 20% except for samples with HQNO concentration at the limit of quantification (IC_80_) when these analyses were performed on different plates or on different days. For the remaining concentration values, the percentage of CV was kept low even when the analyses were performed on different days, which indicates that the assay could quantify with good precision.

**TABLE 3:** Coefficients of variation (CV) of the As389/HHQ-BSA ELISA run in MH culture broth diluted 1/5 using representative concentrations at a low, medium, and high concentration range (IC_20_, IC_50_, and IC_80_)

Reproducibility conditions	IC	Mean	Desv. est.	% CV^*a*^
Interday	20	26.71	3.36	12.6
	50	4.32	0.89	20.6
	80	0.73	0.26	36.2
Interplate	20	25.72	3.91	15.2
	50	2.87	0.56	19.5
	80	0.32	0.12	37.5
Intraplate	20	24.34	3.35	13.8
	50	2.75	0.27	9,9
	80	0.29	0.04	13.9

a The coefficient of variation (CV) was calculated following the equation CV (%) = σ/μ × 100. The results were obtained by measurements performed in either triplicate on the same ELISA plate (intraplate), made on three different days (interday), or by analysis on three different plates (interplate). The concentrations of the replicates, mean, standard deviation, and ICs are expressed in nM. IC: inhibitory concentration; R: replicate; σ: standard deviation; μ: Average.

**FIG 4:**
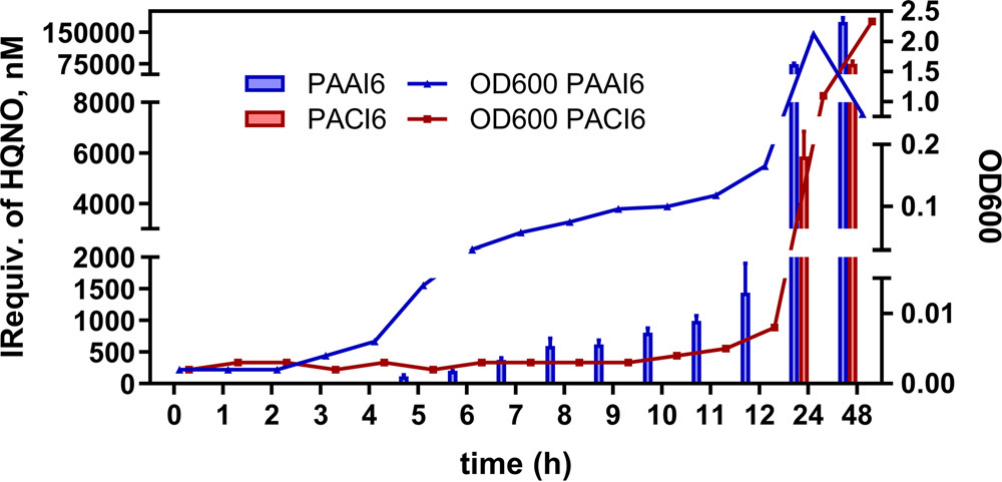
Bacterial growth, expressed as OD_600_ and HQNO immunoreactivity equivalents (IR equivalents) measured in an MH broth medium where P. aeruginosa clinical isolates PAAI6 and PACI6 were cultured. Samples were taken at the selected times and measured using the As389/HHQ-BSA ELISA. Each calibration point was measured in triplicates on the same ELISA plate and the results showed the average and standard deviation of analysis made on two different days.

P. aeruginosa can cause infections with distinct degrees of severity. This pathogen is often producing an overwhelming secretion of virulent factors and immediate host injuries while, in other cases, provokes infections that persist for decades with relative host tolerance which eventually result in decreased lung function and death. Moreover, the pathogenic strains can shift their phenotype between transient and persistent infection seeking long-term survival, evading host immune response, and activating strong defense mechanisms (46). However, during chronic infection, it is also possible to find periods of the more virulent and fulminant disease, termed acute exacerbations. These lifestyle transitions have been studied in terms of extracellular products and common goods release (47). With this scenario, we sought to demonstrate if the release of a virulence factor, such as HQNO, is highly implicated in interspecies interaction and could be related somehow to the severity or stage of the disease. For this purpose, growth curves and HQNO production kinetics of two isolated clinical strains, from an acute (PAAI6) and a chronically infected patient (PACI6), were investigated for 48 h. As it is shown in Fig. 4, the PAAI6 isolate started to release high levels of HQNO after 5 h of growth, while PACI6 did not produce any detectable amount of HQNO before 12 h. To determine that the low levels of the QS target are not only due to the low bacterial viability but to the adaptation of the isolate to the chronic stage of infection isolates from different infection stages were grown at 16 h CFU, and HQNO levels were measured at that time point. As can be observed in Table S6, the data obtained confirmed that the levels of the QS target did not correlate with bacterial growth because similar CF counting numbers correspond to different concentrations of HQNO ranging from 1500 to 12000 nM. The decrease in the optical density at 600 nm (OD_600_) at 48 h of growth of the PAAI6 isolate was interpreted to be caused by the exposure to high concentrations of extracellular products with lytic activity, such as PYO, PQS, or HQNO (48).

**FIG 5:**
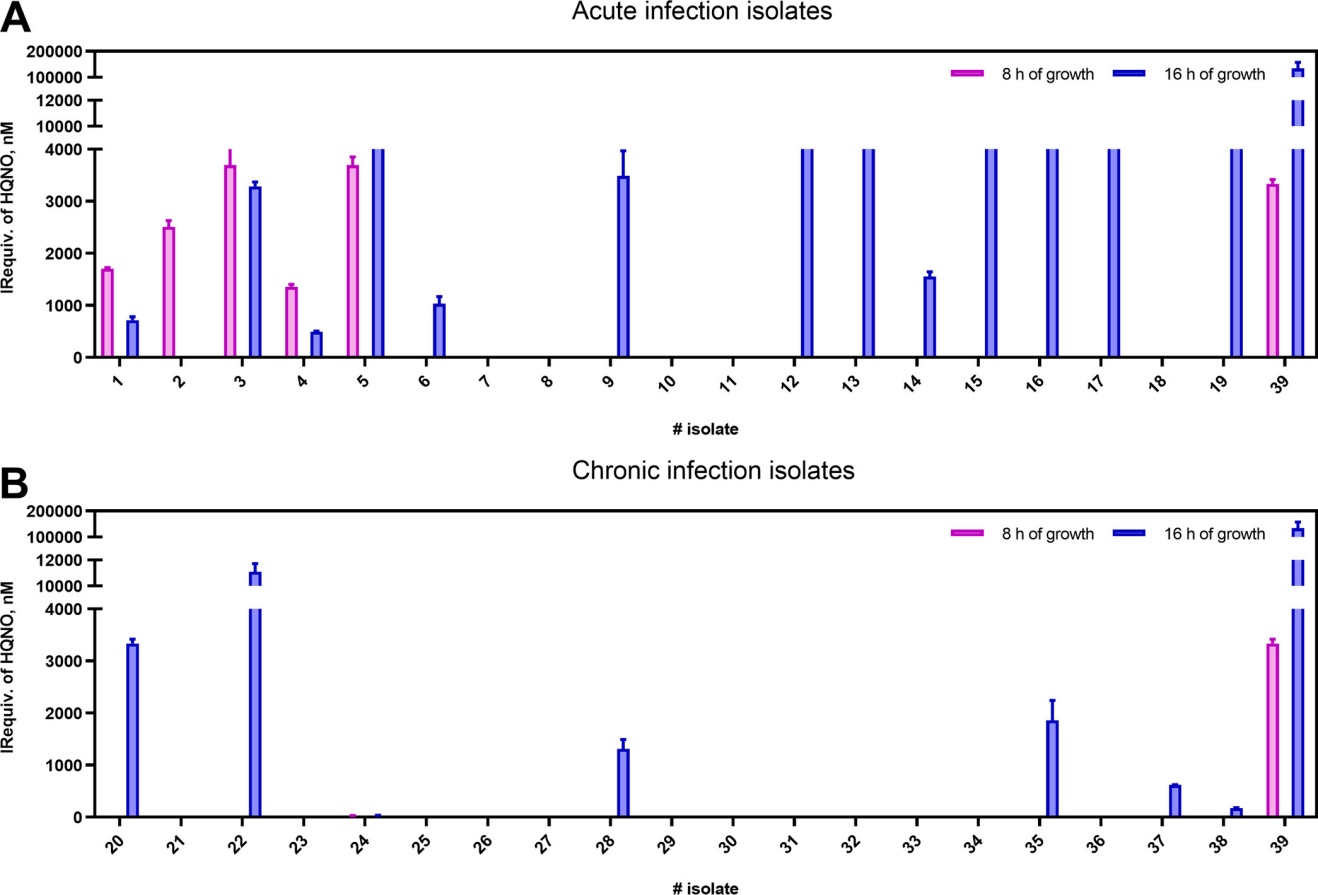
(A) HQNO IR equivalents were recorded from a collection of clinical isolates from patients with acute infection clinical profiles. Samples were grown in MH broth for 8 and 16 h and the aliquots were diluted 5 times with PBS-6.5 before the ELISA analyses. Clinical isolates 1 to 19 were obtained from patients undergoing acute infection and isolate number 39 corresponds to the reference strain PAO1. The reference number of clinical isolates can be found in Table S5. Each calibration point was measured in triplicates on the same ELISA plate and the results showed the average and standard deviation of analysis made on two different days. (B) HQNO IRequiv was recorded from a collection of clinical isolates from patients with chronic infection clinical profiles. Samples were grown in MH broth for 8 and 16 h and the aliquots taken were diluted 5 times with PBS-6.5 before the ELISA analyses. Clinical isolates 20 to 38 were obtained from patients undergoing chronic infection and isolate number 39 corresponds to the reference strain PAO1. The reference number of clinical isolates can be found in Table S5. Each calibration point was measured in triplicates on the same ELISA plate and the results showed the average and standard deviation of analysis made on two different days.

To confirm these results, additional clinical isolates belonging to patients with P. aeruginosa-proven infection at different stages were grown under the same experimental conditions, and the HQNO levels were measured after 8 and 16 h of growth with the As389/HQNO-BSA ELISA (Table S5). The obtained results indicated substantial differences in HQNO production at 8 h between acute and chronic infection isolates differences that were sharpened at 16 h of growth. Thus, as a general trend isolates from an acute infection present higher levels of HQNO than those from a chronic infection. As seen in Fig. 5, there are some cases where undetectable levels of HQNO correspond to an acute infection isolate. To explain these data more, QS biomarkers were analyzed. In this sense, the same group had measured PYO levels in the same bacterial isolates reporting high levels of this virulence factor in most of them, presenting undetectable HQNO levels (49). This would indicate a possible correlation between the levels of both QS molecules, which is a very interesting aspect that should be studied in more detail. Moreover, further experiments will be also addressed to measure HQNO levels directly in clinical samples because, according to the estimations of Barr and coworkers (35, 36), the QSM concentrations in clinical samples should be within the quantitation range of this ELISA. The technology here reported may have great potential for diagnostics, and patient surveillance of the disease progression, helping clinicians in the decision-making process for the management of infected patients.

Antibodies against HQNO, a QS-controlled metabolite and one of the major virulence factors produced by P. aeruginosa, have been developed for the first time. These antibodies were shown to be able to detect HQNO at low concentration levels (LOD of 0.15 ± 0.05 nM and an IC_50_ value of 2.71 ± 0.04 nM) with a quite good specificity in respect to other QS alkylquinolones of the *PqsR* system. The antibodies produced can be excellent tools for the investigation of P. aeruginosa infection to obtain additional knowledge on the progress of the pathogenesis of these bacteria, particularly with respect to the coinfections, which are habitual in chronic patients. Hence, HQNO has been demonstrated to be an inhibitor of the respiratory electron chain of the host cells, but also other usually coexisting bacteria such as S. aureus. Hence, HQNO has been found to exert anti-staphylococcal activity in the lungs of CF patients, where S. aureus is normally outcompeted by P. aeruginosa.

In this paper, we reported the use of these antibodies to develop a microplate-based ELISA for the quantification of HQNO in complex biological samples, aiming at demonstrating the potential of this QS molecule as a biomarker of infection. Preliminary results obtained measuring the profile of release of HQNO of clinical isolates obtained from patients at different disease stages and growth in MH culture broth, the point at the possibility to use this immunochemical tool, not only for diagnosis but to predict the transition between acute and persistent stage in P. aeruginosa infections. Further investigations will be addressed to directly measure such QS-regulated virulence factors on clinical samples, such as sputa, and to perform a complete clinical validation study that demonstrates the potential of HQNO as a biomarker of P. aeruginosa infection and the reliability of the ELISA developed. Finally, the antibodies reported here for the first time can also be used in combination with other biomarkers of infection to develop multiplexed devices to investigate pathogenesis or to develop other immunochemical configurations suitable for point-of-care (PoC) or automated benchtop analyzers.

## MATERIALS AND METHODS

### General methods and instruments.

The general methods and instruments used can be found in Supplemental File 1.

### Synthesis of the immunizing hapten 6-(2-carboxyethyl)-2-heptyl-4-hydroxyquinoline N-oxide (16).

The hapten 6-(2-carboxyethyl)-2-heptyl-4-hydroxyquinoline N-oxide (16) was synthesized from 3-(2-heptyl-4-oxo-1,4-dihydroquinolin-6-yl) propanoate (11) through a three-step synthetic strategy. It followed a similar approach to that described by Woschek et al. (43) for the synthesis of HQNO (Fig. 1). All the intermediates and the final product were characterized by spectroscopic and spectrometric methods (38).

### Synthesis of the bioconjugates HQNO-KLH and HQNO-BSA.

A solution of the HQNO hapten (16) (3.26 mg, 10 μmol) in anhydrous N,N-dimethylmethanamide (DMF) (400 μL) was cooled to 4°C. Subsequently, isobutyl chloroformate (1.56 μL, 12 μmol) and tri-n-butylamine (2.62 μL,11 μmol) were added to the hapten solution and the mixture was stirred for 15 min at 4°C and 30 min at room temperature (RT). Then, 200 μL of the reaction mixture was added over the protein solution (bovine serum albumin [BSA] or keyhole limpet hemocyanin [KLH], 2.5 mg mL^−1^, 1.8 mL in PBS 10 mM) and the mixture was stirred for 2h at RT and overnight at 4°C without agitation. The bioconjugates were purified by dialysis against 0.5 mM PBS (5 × 5 L) and Milli-Q water (1 × 5 L), and freeze-dried at −80°C. A small fraction (20 μL) of the HQNO-BSA was kept for MALDI-TOF analysis, rendering a hapten density of 21 haptens per molecule of BSA (Table S1).

### ELISA.

**(i) As389/HHQ-BSA ELISA.** Microtiter plates were coated with a solution of the HHQ-BSA bioconjugate in coating buffer (0.25 μg mL^−1^, 100 μL/well) overnight at 4°C and covered with an adhesive plate sealer. The day after, the plates were washed with PBST (4 × 300 μL/well) and solutions of HQNO standards (2 μM to 0.13 nM in PBST, 50 μL/well) followed by the As389 (Diluted 1/16000 in PBST, 50 μL/well) were added and the microplates left without agitation 30 min at RT. The plates were washed as before and a solution of goat anti-rabbit IgG-HRP (1/6000 in PBST) was added (100 μL/well) and incubated for 30 min more at RT. The plates were washed again, and the substrate solution was added (100 μL/well) and then left for 30 min at RT in the dark. The enzymatic reaction was stopped by adding 4N H_2_SO_4_ solution (50 μL/well) and the absorbance read at 450 nm.

**(ii) Immunoassay performance evaluation.** Performance of the assays was evaluated through the modification of different physicochemical parameters (competence time, incubation time, pH, ionic strength, presence of a surfactant [% Tween 20], and solubility with the addition of organic solvents) in the competition step.

**(iii) Cross-reactivity studies.** Standard solutions of the main alkyl quinolones from Pseudomonas aeruginosa (HHQ, PQS, and HQNO) were prepared (1 pM to 10 μM in phosphate buffered saline with Tween 20 [PBST]) and measured with the ELISA following the procedure described above. The standard curves obtained were fitted to the four-parameter equation mentioned above and the IC_50_ value was used to calculate the cross-reactivity according to the following equation: CR (%) = IC_50_(Cross reactant)/IC_50_(Analyte) × 100.

### Implementation of the ELISA to the analysis of clinical isolates.

See Supplemental File 1 for a detailed procedure of clinical isolates growth procedure.

**(i) Matrix effect and accuracy studies.** MH culture medium was diluted (1:2, 1:5, 1:10, and 1:20), and used to prepare HQNO standard calibration curves and to compare them with the standard curves prepared in PBS-6.5. Subsequently, the dilution providing the best ELISA parameters was selected and the concentrations of CA and AS dilution were adjusted to minimize matrix interferences. For the accuracy studies, blind spiked samples prepared in diluted MH culture broth were measured using the above reported ELISA. The samples were measured in triplicates and the experiment was repeated on three different days.
