# Supplementary material for: An Immunochemical Approach to Detect the Quorum Sensing-Regulated Virulence Factor 2-Heptyl-4-Quinoline N-Oxide (HQNO) Produced by Pseudomonas aeruginosa Clinical Isolates
Source: Microbiol Spectr. 2022 Jul 25;10(4):e01073-21. doi: 10.1128/spectrum.01073-21 (PMC9431570; doi:10.1128/spectrum.01073-21)
Supplement: Supplemental file 1 — Supplemental material. Download spectrum.01073-21-s0001.pdf, PDF file, 0.8 MB [file spectrum.01073-21-s0001.pdf]

Supporting information

An Immunochemical Approach to detect the QS Regulated Virulence Factor 2-Heptyl-4-Quinoline N-Oxide (HQNO) produced by *Pseudomonas* aeruginosa Clinical Isolates

Enrique-J. Montagut[A,B], Juan Raya[A,B], M.-Teresa Martin-Gomez[C,D] , Lluïsa Vilaplana[A,B], Barbara Rodriguez-Urretavizcaya[A,B] and M.-Pilar Marco[A,B]*

A. Nanobiotechnology for diagnostics (Nb4D), Department of Surfactants and Nanotechnology, Institute for Advanced Chemistry of Catalonia (IQAC) of the Spanish Council for Scientific Research (CSIC), Spain.

B. CIBER de Bioingeniería, Biomateriales y Nanomedicina (CIBER-BBN), Jordi Girona 18-26, 08034 Barcelona, Spain

C. Microbiology Department, Vall d'Hebron University Hospital (VHUH), Barcelona, Spain

D. Genetics and Microbiology Department, Universitat Autònoma de Barcelona (UAB), Barcelona, Spain

*To whom correspondence should be sent:

M.-Pilar Marco
Nanobiotechnology for diagnostics (Nb4D) group
Department of Surfactants and Nanotechnology
IQAC-CSIC
Jordi Girona 18-26, 08034 Barcelona, Spain
Spain

Phone: + 34 93 4006184
FAX: + 34 93 2045904
E-mail: pilar.marco@cid.csic.es

MATERIALS AND INSTRUMENTS:

Chemistry. The chemicals used in the synthesis of the haptens were obtained from Aldrich Chemical Co. (Milwaukee, WI, USA), Sigma Chemical Co. (St. Louis, MO, USA) or Acros Organics B.V.B.A. (Morris Plains, NJ, USA). Thin-layer chromatography (TLC) was performed on 0.25 mm, pre-coated silica gel 60 F254 aluminium sheets (Merck, Darmstadt, Germany). ^{1}H and ^{13}C NMR spectra were obtained with a Varian Mercury-400 spectrometer (400 MHz ^{1}H and 101 MHz for ^{13}C). Liquid chromatography/electrospray ionization/mass spectrometry (LC/ESI/MS) was performed in a Waters (Milford, MA, USA) model composed by an Acquity UPLC system directly interfaced to a Micromass LCT Premier XE MS system equipped with an ESI LockSpray source for monitoring positive and negative ions. Data were processed with MassLynx (V 4.1) software (Waters).

Immunochemistry. Chemicals and biochemicals were obtained from Aldrich Chemical Co. (Milwaukee, WI, USA) and from Sigma Chemical Co. (St. Louis, MO, USA). The HHQ (2-heptyl-4-quinolone) hapten used in this study was prepared following a similar synthetic procedure as that described by Reen et al.[41] and conjugated to BSA (HHQ-BSA, hapten density 13). The stock solutions of the alkylquinolones (HHQ, PQS, HQNO) used as standards were prepared in DMSO at 10 mM and stored at -20ºC, then transferred to 4ºC prior to their use. Purification of the bioconjugates was carried out in ÄKTA Prime Plus using 2 HiTrap desalting columns both from GE Healthcare (Chicago, IL, USA) or either by dialysis using Spectra/Por membranes from Spectrumlabs (Piraeus, Greece, EU) with molecular weight cut-off of 12-14 kDa. The matrix-assisted laser desorption ionization time-of-flight mass spectrometer (MALDI-TOF-MS) was a Bruker Autoflex III Smartbeam spectrometer (Billerica, Massachusetts). Hapten densities of the bioconjugates were calculated by MALDI-TOF-MS by comparing the molecular weight recorded on the MALDI spectra of the native proteins to that of the HQNO-BSA bioconjugates. For this purpose, the bioconjugates were mixed with the freshly prepared matrix ((trans-3,5-dimethoxy-4-hydroxycinnamic acid, 10 mg mL^{-1} in 70:30 ACN/H2O, 0.1% HCOOH) following the ''sandwich'' sample preparation method. The bioconjugate aliquot was diluted ½ using CAN (0.2% HCOOH). According to it, the matrix (2 µL) is deposited on the MALDI plate and dried, followed by the bioconjugate solution (2 µL, 2 to 5 mg mL^{-1} in 1:1 ACN/H2O, 0.1% HCOOH), allowed to dry again and finally, the matrix solution (2 µL) was added over again. The resulting dried spot was then analyzed by MALDI-TOF-MS. Hapten densities were calculated through the equation: [MW(conjugate) − MW(native protein)]/[MW(hapten)-MW(lost atoms)]. The pH and the conductivity of all buffers and solutions were measured with a pH-meter pH 540 GLP and a

conductimeter LF 340, respectively (WTW, Weilheim, Germany). Polystyrene microtiter plates used for the ELISAs were purchased from Nunc (Maxisorp, Roskilde, Denmark). Dilution plates were purchased from Nirco (Barberà del Vallés, Spain). Washing steps were performed on a Biotek ELx465 (Biotek Inc.). Absorbances were read on a Thermo Scientific MultiSkan GO (Thermo Fisher Scientific, Waltham, MA, USA) at a single wavelength mode (450 nm). The competitive curves were analyzed with a four-parameter logistic equation using the software GraphPad Prism 7.0 (GraphPad Software Inc., San Diego, CA, USA) according to the following formula: $y = B(A-B)/[1 - (x/C)^D]$, where A is the maximum absorbance, B is the minimum absorbance, C is the concentration producing 50% of the maximal absorbance, and D is the slope at the inflection point of the sigmoid curve. Unless otherwise indicated, the data presented correspond to the average of at least two well replicates.

Buffers. Unless otherwise indicated, phosphate buffer saline (PBS) corresponds to 10mM phosphate buffer and 0.8% saline solution (pH 7.5). The buffer of the assay selected after optimization of the physicochemical parameters was PBS-6.5 (PBS adjusted to pH 6.5 with 1N HCl). Coating buffer is a 50 mM bicarbonate-carbonate buffer (pH 9.6). PBST is PBS with 0.05% Tween 20 (pH 7.5). Citrate buffer corresponds to a 40 mM sodium citrate solution (pH 5.5). The substrate solution contains 0,01% of 3,3',5,5'- tetramethylbenzidine (TMB) and 0.004% H_2O_2 prepared in citrate buffer. Borate buffer is 0.2 M sodium borate/boric acid (pH 8.7). All buffers were prepared using ultra-pure Milli-Q® water with a resistivity between 16-18 MΩ cm.

Polyclonal antisera (PAb). Three female New Zealand white rabbits weighing 1–2 kg were immunized with HQNO-KLH following established protocols in the research group. Immunizations were carried out in the animal facility of the Research and Development Center (CID) of the Spanish Research Council (CSIC) Registration Number: B9900083, employing approved procedures that avoid unnecessary treatments and minimize suffering of the animals. The protocol used in accordance with the institutional guidelines under a license from the local government (DAAM 7463) and approved by the Institutional Animal Care and Use Committee at the CID-CSIC. The antisera (As) obtained were named As388, As389 and As390. The animals were exsanguinated after 6 immunizations, and the final blood was collected in vacutainer tubes provided with a serum separation gel. Antisera were obtained by centrifugation at 4 °C for 10 min at 10 000 rpm, then stored at −80 °C in the presence of preservative 0,02% sodium azide. The antibody titer was assessed during the immunization process through non-competitive indirect ELISA. Microtiter plates were coated with a fixed concentration of HQNO-BSA conjugate (1 mg mL^{-1}) and the avidity of the produced antibodies was measured by preparing serial dilutions of the corresponding As.

IMMUNOGEN AND COATING ANTIGEN PREPARATION:

Table S1. Data on the bioconjugation yield and hapten densities of the HQNO bioconjugates.

	Quantity (mg)	Yield (%)	Hapten density
**HQNO-BSA**	3.38	67	21
**HQNO-KLH**	3.34	67	-

Hapten densities of BSA conjugates were calculated from MALDI-TOF-MS analysis. KLH conjugate could not be analyzed by MALDI-TOF-MS because of the large molecular weight. For this purpose, BSA ans KLH bioconjugates were prepared in paralell, under exactly the same conditions. The data obtained with the corresponding BSA bioconjugates was used as bioconjugation control.

COMPETITIVE ASSAY SELECTION:

Non-competitive indirect two-dimensional titration experiments. Titration experiments in non-competitive indirect ELISA format were performed in order to establish the best conditions of As dilution and concentration of coating antigen (CA) for the competitive quantification assays. With this aim, the dilutions of antisera (1/1000 to 1/64000, 100 µL/well) were added to Microtiter plates, which were previously coated with BSA competitors (5 µg mL^{-1} to 5 ng mL^{-1}, coating buffer, 100 µL/well) and washed with PBST (4 x 300 µL/well), and the binding measured following the protocol described in section 2.4.2. The combination of As dilution and CA concentration producing around 0.7-1.2 units of absorbance was selected.

Table S2. Analytical parameters of the competitive indirect ELISA for the detection of HQNO that were selected after bi-dimensional titration experiments.

	**As 388; 1/8000**	**As 389; 1/4000**
**[CA] (µg/ml)**	HHQ-BSA; 0.25	HHQ-BSA; 0.25
Bottom	0,15	0,23
Top	2,62	1,80
Hill Slope	-0,67	-0,55
IC_{50}	4824	46,9
R^2	0,994	0,999

PHYSICOCHEMICAL PARAMETERS OPTIMIZATION:

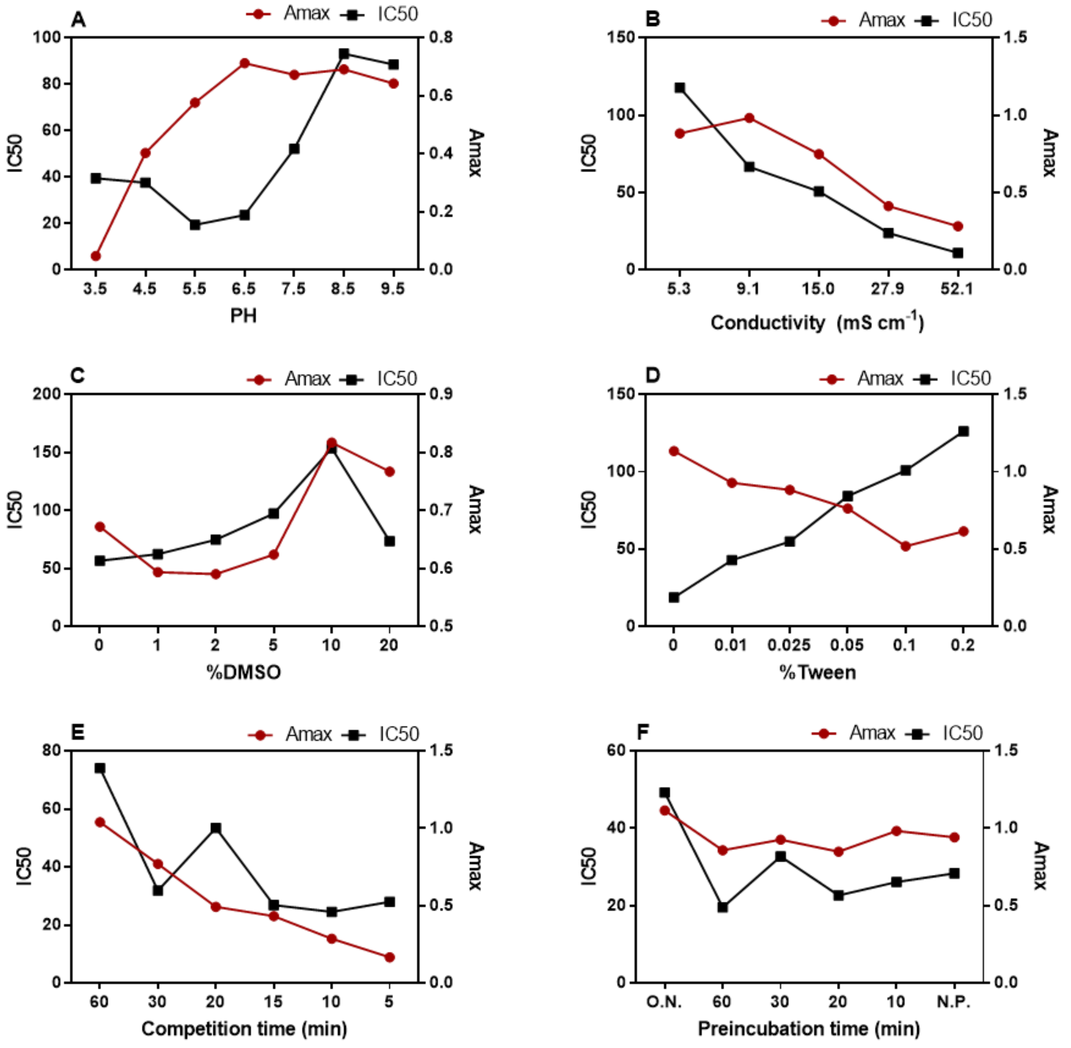

Figure. S1. As389/ HHQ-BSA ELISA performance in the physicochemical parameters optimization study. The selection of the most appropriate conditions (**Table S3.**) was based on the variations in Amax, IC50 and slope values (not shown) of the generated calibration curves providing better signal/noise ratio, detectability and sensitivity. The studied parameters were **A.** pH **B.** Ionic Strength **C.** % organic solvent (DMSO) **D.** % Tween20 **E.** competition time **F.** preincubation time. All the studies were performed by varying the composition of the buffer used in the competitive step or the antibody detection times. The values of the IC50 are expressed in nM. Eventually, the conditions providing better features were evaluated again separately and in conjunction.

Table S3. Physicochemical parameters selected for the As389/HHQ-BSA.

	**As389 HHQ-BSA**
**As dilution**	1/8000
**[Competitor] (µg mL^{-1})**	0.250
**pH**	6.5
**Conductivity (mS cm^{-1})**	15
**Tween 20 (%)**	0
**Competition time (min)**	30
**Preincubation time (min)**	0
**Organic solvent (%)**	0

The parameters improving the features of the assay were assessed separately and in conjunction.

ELISA EVALUATION:

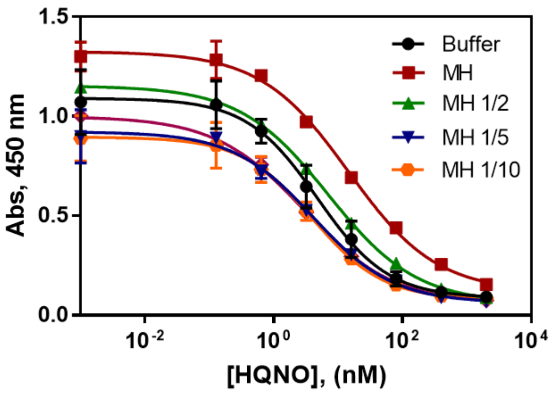

Figure S2. Matrix effect of the MH broth undiluted and diluted 2, 5, 10 and 20 times with PBST on the As389/ HHQ-BSA ELISA. The calibration curves were run using the conditions established for the assay in PBS-6.5. Modification of the assay conditions allowed achieving similar immunoassay features as when the assay was run in buffer (see Figure 2). The results shown are the average and standard deviations of analysis made on two different days measured by duplicates each day.

HQNO PRODUCTION IN CLINICAL ISOLATES:

Samples. *Clinical isolates* were obtained from lower respiratory tract samples, mainly sputum specimens, of patients diagnosed of acute or chronic respiratory infections. Clinical samples were cultured in MacConkey agar, Blood agar and Chocolate agar, and incubated for up to 5 days at 37°C. *P. aeruginosa* isolates were selected and stored frozen at -20 ºC. Prior the analyses, *P. aeruginosa* isolates were cultured overnight at 37ºC in blood agar plates. The day after, a portion of the grown bacteria was diluted in MH culture broth (3 mL) and shaken at 37ºC. When the optical density at 600 nm (OD_{600}) reached a value of 0.2-0.3, a dilution (1/1000 in 20 mL of MH) was performed and the resulting solution shaken at 37ºC. For experiments aimed at analyzing the HQNO release profile, aliquots were taken at selected times for measurement of the OD_{600}andCFUs (Colony Forming Units), and to quantify HQNO immunoreactivity equivalents (IR equiv.) by ELISA. For CFU counting 10 μL of different dilutions of the isolate time point cultures were plated on blood agar plates that were grown an o/n at 37 ^{o}C. The day after, colonies were counted for the dilutions giving between 10 and 100 isolated colonies. On experiments aimed to compare HQNO IR equiv. of the different clinical isolates the same experimental procedure was used, yet the aliquots were taken just at a selected time of 8 h. HQNO concentrations, measured by ELISA are expressed as HQNO IR equiv., due to the potential specific interferences caused by other alkylquinolones present in the culture media.

Table S4. Colony Forming Units (CFUs) calculated using the formula described by Dong-Ju Kim et al[1]. using the optical density measured at 600 nm (OD_{600}) for clinical isolates PAAI6 and PACI6.

t (h)	OD_{600} acute	CFUs acute	OD_{600} chronic	CFUs chronic
**0**	0.002	4,4E+06	0.002	4,4E+06
**1**	0.003	4,6E+06	0.002	4,4E+06
**2**	0.003	4,6E+06	0.002	4,4E+06
**3**	0.004	4,8E+06	0.002	4,4E+06
**4**	0.006	5,2E+06	0.003	4,6E+06
**5**	0.014	6,8E+06	0.002	4,4E+06
**6**	0.031	1,0E+07	0.003	4,6E+06
**7**	0.058	1,6E+07	0.003	4,6E+06
**8**	0.075	1,9E+07	0.003	4,6E+06
**9**	0.096	2,3E+07	0.003	4,6E+06
**10**	0.100	2,4E+07	0.004	4,8E+06
**11**	0.118	2,8E+07	0.005	5,0E+06
**12**	0.165	3,7E+07	0.008	5,6E+06
**24**	2.125	-	1.096	2,2E+08
**48**	0.796	1,6E+08	2.333	-

Table S5: Clinical isolates reference number and concentration of HQNO measured with the developed As389/ HQNO-BSA ELISA.

#Isolate	#Ref	Infection type	[HQNO]/nM at 8 h	Desv. Est. (nM)	[HQNO]/nM at 16 h	Desv. Est. (nM)
**1**	**PAAI1**	**Acute**	1703.9	23.6	718.02	63.56
**2**	**PAAI2**	**Acute**	2507.0	124.7	LLOQ	--
**3**	**PAAI3**	**Acute**	3693.4	391.8	3285.15	86.99
**4**	**PAAI4**	**Acute**	1356.4	47.3	499.15	8.09
**5**	**PAAI5**	**Acute**	3691.7	158.8	5407.05	765.83
**6**	**PAAI6**	**Acute**	595.4	120.5	1030.67	140.51
**7**	**PAAI7**	**Acute**			LLOQ	-
**8**	**PAAI8**	**Acute**			LLOQ	-
**9**	**PAAI9**	**Acute**			3487.88	482.52
**10**	**PAAI10**	**Acute**			LLOQ	-
**11**	**PAAI11**	**Acute**			LLOQ	-
**12**	**PAAI12**	**Acute**			6935.99	1149.18
**13**	**PAAI13**	**Acute**			7207.75	1072.62
**14**	**PAAI14**	**Acute**			1558.40	86.91
**15**	**PAAI15**	**Acute**			7060.90	1014.54
**16**	**PAAI16**	**Acute**			9573.53	107.70
**17**	**PAAI17**	**Acute**			6767.04	248.32
**18**	**PAAI18**	**Acute**			LLOQ	-
**19**	**PAAI19**	**Acute**			8420.56	1211.08
**20**	**PACI1**	**Chronic**	LLOQ	-	LLOQ	-
**21**	**PACI2**	**Chronic**	LLOQ	-	11090.99	631.77
**22**	**PACI3**	**Chronic**	1.86	0.7	LLOQ	-
**23**	**PACI4**	**Chronic**	LLOQ	-	10.55	0.69
**24**	**PACI5**	**Chronic**	20.0	12.2	LLOQ	-
**25**	**PACI6**	**Chronic**	LLOQ	-	LLOQ	-
**26**	**PACI7**	**Chronic**			LLOQ	-
**27**	**PACI8**	**Chronic**			173.08	10.92
**28**	**PACI9**	**Chronic**			LLOQ	-
**29**	**PACI10**	**Chronic**			LLOQ	-
**30**	**PACI11**	**Chronic**			LLOQ	-
**31**	**PACI12**	**Chronic**			LLOQ	-
**32**	**PACI13**	**Chronic**			LLOQ	-
**33**	**PACI14**	**Chronic**			LLOQ	-
**34**	**PACI15**	**Chronic**			1859.45	382.66
**35**	**PACI16**	**Chronic**			29.58	8.49
**36**	**PACI17**	**Chronic**			621.78	3.42
**37**	**PACI18**	**Chronic**			1309.40	182.63
**38**	**PAO**	**Reference**	3336.2	81.7	133526.25	23481.07

The clinical isolates were grown 8 and 16 hours in MH culture media at 37 ºC following the protocol described in the main article.

Table S6: Levels of HQNO measured with the developed As389/ HQNO-BSA ELISA and CFU/mL counting after 16 hours of growth of different clinical isolates.

IR equiv. of HQNO, nM	CFU/mL
2311.35	$2.00 \cdot 10^{9}$
8796.76	$1.70 \cdot 10^{9}$
6402.15	$4.00 \cdot 10^{9}$
7951.24	$1.30 \cdot 10^{10}$
12493.42	$1.40 \cdot 10^{10}$
6803.22	$2.20 \cdot 10^{10}$
1512.22	$2.40 \cdot 10^{10}$

Supporting information references:

1. Dong-ju Kim,. Relation of microbial biomass to counting units for Pseudomonas aeruginosa. *African J. Microbiol. Res.* **6**, 4620–4622 (2012).
